# Supplementary material for: Comparative genomics provides new insights into the diversity, physiology, and sexuality of the only industrially exploited tremellomycete: Phaffia rhodozyma
Source: BMC Genomics. 2016 Nov 9;17:901. doi: 10.1186/s12864-016-3244-7 (PMC5103461; doi:10.1186/s12864-016-3244-7)
Supplement: Additional file 6: — List of orphan genes with links to PFAM (related to Additional file 1: Table S1). (ZIP 1428 kb) [file 12864_2016_3244_MOESM6_ESM.zip › PFAM_UNIQUE/G03335_P.html]

#                                                                            --- full sequence --- -------------- this domain -------------   hmm coord   ali coord   env coord
  
# target name        accession   tlen query name           accession   qlen   E-value  score  bias   #  of  c-Evalue  i-Evalue  score  bias  from    to  from    to  from    to  acc description of target
  
#------------------- ---------- ----- -------------------- ---------- ----- --------- ------ ----- --- --- --------- --------- ------ ----- ----- ----- ----- ----- ----- ----- ---- ---------------------
  
G03335\_P             -            117 BLUF                 PF04940.7     93   0.00029   19.2   0.6   1   2       2.2   2.7e+03   -3.1   0.2    13    13    31    31    15    46 0.48 - Sensors of blue-light using FAD  
G03335\_P             -            117 BLUF                 PF04940.7     93   0.00029   19.2   0.6   2   2   3.4e-07    0.0004   18.7   0.0     2    37    70   106    69   111 0.89 - Sensors of blue-light using FAD  
G03335\_P             -            117 DUF4614              PF15391.1    182       2.7    6.4   5.8   1   1    0.0054       2.9    6.3   5.8    15    60     7    40     1    79 0.46 - Domain of unknown function (DUF4614)  
G03335\_P             -            117 DUF566               PF04484.7    313       1.5    7.0   5.0   1   1    0.0055       1.4    7.0   5.0    38    83     3    46     1   113 0.47 - Family of unknown function (DUF566)  
G03335\_P             -            117 EB1\_binding          PF05937.6    174       4.8    6.2  11.3   1   1   0.00095       5.7    6.0  11.3    94   155     7    68     1    81 0.78 - EB-1 Binding Domain  
G03335\_P             -            117 FAM117               PF15388.1    318       7.8    4.3   8.9   1   1     0.014       8.6    4.1   8.9   181   220     4    42     1    87 0.41 - Protein Family FAM117  
G03335\_P             -            117 NCD2                 PF04905.8    164       0.2   10.1   6.1   1   1   0.00063       1.9    6.9   6.1    23    81    51   110     2   115 0.73 - NAB conserved region 2 (NCD2)  
G03335\_P             -            117 Opi1                 PF08618.5    427       2.2    5.8   6.7   1   1     0.001         2    5.9   6.7   146   195    11    57     2   105 0.75 - Transcription factor Opi1  
G03335\_P             -            117 Period\_C             PF12114.3    195       4.9    5.4   8.2   1   1     0.008       5.3    5.3   8.2    44    77    11    44     1    78 0.64 - Period protein 2/3C-terminal region  
G03335\_P             -            117 SOG2                 PF10428.4    445       1.2    6.4   5.3   1   1    0.0056       1.2    6.4   5.3   184   279     6    99     1   113 0.46 - RAM signalling pathway protein  
G03335\_P             -            117 Sporozoite\_P67       PF05642.6    727       5.4    3.4   7.3   1   1     0.036       5.8    3.3   7.3   276   322     5    52     1    75 0.57 - Sporozoite P67 surface antigen  
G03335\_P             -            117 SSP160               PF06933.6    756       2.8    4.5   9.5   1   1     0.012       2.6    4.6   9.5   105   135    10    40     2   103 0.56 - Special lobe-specific silk protein SSP160
